# Editorial: Global excellence in inflammatory diseases: Latin America 2021

**DOI:** 10.3389/fimmu.2023.1278212

**Published:** 2023-09-27

**Authors:** Roberto César Pereira Lima-Júnior, José C. Crispín, Gerly Anne Castro Brito

**Affiliations:** ^1^ Center for Drug Research and Development, Department of Physiology and Pharmacology, Faculty of Medicine, Federal University of Ceara, Fortaleza, Brazil; ^2^ Department of Immunology and Rheumatology, Instituto Nacional de Ciencias Médicas y Nutrición Salvador Zubirán, Mexico City, Mexico; ^3^ Tecnologico de Monterrey, Escuela de Medicina y Ciencias de la Salud, Monterrey, Mexico; ^4^ Department of Morphology, Faculty of Medicine, Federal University of Ceara, Fortaleza, Brazil

**Keywords:** inflammation, immunogenic cell death, *Clostridioides difficile*, intestine, infection, arthritis

The immune system acts as a double-edged sword. Depending on the context, it may protect from disease, or contribute to its expression. For example, chronic inflammation promotes the development of some malignancies and neutrophils may facilitate metastatic spread of cancer cells (1). Conversely, the immune system detects and destroys malignant cells and pathogens and orchestrates tissue repair when cellular injury occurs (2). The complex interplay of its protective and pathogenic capacities determines the clinical expression of infectious, inflammatory, and malignant diseases. The present Research Topic contains five reports that highlight these aspects of immune function and illustrate how the immune system can contribute to disease.

The study by Florêncio et al. examined the capacity of chromomycins, a type of marine-derived antibiotics, to induce immunogenic cell death (ICD) in melanoma. Using an elegant experimental approach, the authors demonstrated that chromomycin A5 could trigger endoplasmic reticulum stress, autophagy, and apoptosis in B16-F10 melanoma cells. Chromomycin-stimulated cells released ICD-related damage-associated molecular patterns, such as HMGB1 (high mobility group box 1), ATP, and calreticulin (CRT). These effects led to the activation of CD11b^+^CD11c^+^ dendritic cells with high expression of costimulatory (i.e. CD80 and CD86) and MHC-II molecules. As a result, chromomycin A5 reduced tumor growth in an *in vivo* murine model. Notably, vaccination of mice with chromomycin A5-treated B16-F10 cells, induced T cell activation and protection against tumor challenge with viable B16-F10 cells. These results are relevant, as only a few chemotherapeutic agents have been shown to elicit strong ICD. Therefore, the molecule described by Florêncio et al. could help reverse the immunosuppressive microenvironment produced by viable tumors and thus improve the sensitivity to immunotherapy (3).

One of the main caveats of anticancer chemotherapeutic drugs is their lack of specificity, and consequent damage to non-tumor cells. Intestinal mucositis is a common side effect of 5-fluorouracil (5-FU)-based anticancer regimens. The treatment for this condition is mainly symptomatic and not consistently effective (4). The study by Barbosa et al. shed light on the protective effects of *Lacticaseibacillus casei* (*L. casei*), an orally administer probiotic, as a promising option for treating mucositis. Mice gavaged with *L. casei* showed a pronounced reduction in 5-FU-associated intestinal damage. The probiotic increased fecal lactic acid bacteria and reduced the production of inflammatory mediators, such as toll-like receptor 4 (TLR4), tumor necrosis factor-alpha (TNF-α), interleukin-1β (IL-1β), IL-6, and inducible nitric oxide synthase (iNOS). Additionally, administration of *L casei* increased the expression of mucosal integrity markers, such as mucin-2 and tight junction intercellular barrier components ZO-1 and occludin. The benefits of probiotics have been investigated mainly as therapeutic alternatives for chemotherapy-associated intestinal mucositis (5) and the study by Barbosa et al. represents a step toward a better clinical management of mucositis.

Gut microbiome imbalances, frequently found in patients receiving antibiotics, chemotherapy, and radiotherapy, are primary factors contributing to *Clostridioides difficile* infection (6). Loureiro et al. and Costa et al. investigated how *C. difficile* toxins A (TcdA) and B (TcdB) trigger cell death and expression of proinflammatory mediators in enteric glial cells. Loureiro et al. demonstrated that TcdA and TcdB-challenged enteric glial cells increasing the expression of pannexin-1 (Panx1) channels. These transmembrane proteins are known to release adenosine triphosphate (ATP), which activates P2X7 receptors (P2X7R) (7). Interestingly, the toxins also upregulated the expression of P2X7R. Experiments using pharmacological inhibition confirmed the role of PANX1 and P2X7R in TcdA and TcdB-mediated cell death and IL-6 production as part of *C. difficile* infection mechanisms. Adenosine is a purine nucleoside that may derive from ATP degradation and mitigate inflammatory responses (8). Remarkably, Costa et al. showed that upregulation of adenosine receptors A2A and A3, and downregulation of A2B, in enteric glial cells, and downregulation of A2B in intestinal tissues elicits a protective response against *C. difficile* toxins. Although TcdA and TcdB induce protein kinase A (PKA) and CREB phosphorylation to cause enteric glial cell death, the study showed that the anti-apoptotic effects of adenosine receptors were independent of these signaling pathways. The intricate mechanisms that drive *C. difficile* infection are not fully understood yet, but identifying pathogenic signaling opens an avenue for effective pharmacological targeting. The studies by Loureiro et al. and Costa et al. provide further contributions.

Inflammatory conditions can be modulated by inhibiting the proinflammatory network or enhancing anti-inflammatory mechanisms. Lipoxin A_4_ (LXA_4_) is an example of a specialized pro-resolution lipid mediator (9). Saraiva-Santos et al. tested this concept in a titanium dioxide (TiO_2_) arthritis animal model, which mimics prosthesis-induced joint inflammation and pain. Mice supplemented with LXA_4_ showed reduced mechanical and thermal hyperalgesia, histopathological damage, edema, and recruitment of leukocytes without signs of systemic toxicity. The mechanism involved enhancing antioxidant mechanisms and regulating transcription factors, such as nuclear factor kappa B (NF-κB) and nuclear factor erythroid 2-related factor 2 (Nrf2). Additionally, the lipid mediator downregulated the pain-associated transient receptor potential channels, TRPV1 and TRPA1, in the dorsal root ganglion pointing out its antinociceptive effect. The study by Saraiva-Santos et al. contributes to our understanding of how resolution mechanisms could potentially develop novel therapeutics for treating inflammatory conditions.

The collection presented in this Research Topic delves into different mechanisms of how the immune system can be modulated to improve malignant and inflammatory conditions. It illustrates how the mechanisms that underlie inflammatory responses in the gut due to cancer chemotherapy toxicity or infectious diseases could be adequately explored to identify potential pharmacological targets. The significance of these findings must be tested in human pathology in the clinical setting to be fully validated.

## Author contributions

RL: Writing – original draft. JC: Writing – review & editing. GB: Writing – review & editing.
